# Transient receptor potential ion-channel subfamily V member 4: a potential target for cancer treatment

**DOI:** 10.1038/s41419-019-1708-9

**Published:** 2019-06-24

**Authors:** Suyun Yu, Shuai Huang, Yushi Ding, Wei Wang, Aiyun Wang, Yin Lu

**Affiliations:** 1 Jiangsu Key Laboratory for Pharmacology and Safety Evaluation of Chinese Materia Medica, School of Pharmacy, Nanjing University of Chinese Medicine, Nanjing, P. R. China; 2 Jiangsu Collaborative Innovation Center of Traditional Chinese Medicine Prevention and Treatment of Tumor, Nanjing University of Chinese Medicine, Nanjing, P. R. China

**Keywords:** Cancer, Biomarkers

## Abstract

The transient receptor potential ion-channel superfamily consists of nonselective cation channels located mostly on the plasma membranes of numerous animal cell types, which are closely related to sensory information transmission (e.g., vision, pain, and temperature perception), as well as regulation of intracellular Ca^2+^ balance and physiological activities of growth and development. Transient receptor potential ion channel subfamily V (TRPV) is one of the largest and most diverse subfamilies, including TRPV1–TRPV6 involved in the regulation of a variety of cellular functions. TRPV4 can be activated by various physical and chemical stimuli, such as heat, mechanical force, and phorbol ester derivatives participating in the maintenance of normal cellular functions. In recent years, the roles of TRPV4 in cell proliferation, differentiation, apoptosis, and migration have been extensively studied. Its abnormal expression has also been closely related to the onset and progression of multiple tumors, so TRPV4 may be a target for cancer diagnosis and treatment. In this review, we focused on the latest studies concerning the role of TRPV4 in tumorigenesis and the therapeutic potential. As evidenced by the effects on cancerogenesis, TRPV4 is a potential target for anticancer therapy.

## Facts

1. TRPV4 is a broadly expressed, nonselective calcium permeant cation channel that perform an important role in regulating the Ca^2+^ influx in the cells in which they are expressed.
2. TRPV4 is a thermosensor, activated by temperatures greater than 24–27 °C, also can be activated by osmotic, mechanical, and chemical cues.
3. TRPV4 is constitutively expressed and capable of spontaneous activity in the absence of agonist stimulation, which suggests that it serves important physiological functions.

## Open questions

1. What is the underlying cause of TRPV4 expression levels in different types of cancer?
2. Whether it is possible to lower the toxicity and high-efficiency TRPV4 inhibitors from natural products to treat various diseases?
3. How to inhibit the development of cancer by targeting TRPV4 of tumor cells without affecting the function of normal cells?

## Introduction

Malignant tumors are still the primary health problems that plague people^1^, which are clinically treated mainly through chemotherapy, biological therapy, radiotherapy, and surgical resection^2^. However, chemotherapy usually causes severe side effects and proneness to drug resistance^2^. Therefore, researchers have endeavored to find molecular targeted drugs which are less toxic and more efficient. Targeted therapy using drugs which designed based on antitumor targets interferes with or even blocks the physiological activities of tumors^3^. Drugs that target cancer cell membrane receptors have the advantages of high affinity and recognition efficiency^4^. For instance, drugs targeting epidermal growth factor receptors and some other cell membrane receptors have been clinically used^5^. Several important cellular functions are related to transmembrane potentials and lie under the control of ion channels. The role of ion channels play in connecting the intracellular to the extracellular environment and controlling almost any cellular function makes ion channels ideal potential therapeutic targets. Nowadays, increasing interest has been given to ion channels as potential drug targets in many cancer conditions^6,7^. Many observations found that some cancer cell lines display unusual ion channel expression, and the altered ion transport may play an important role in human cancer progression. When ion channels open, certain ion species can pass through which affects such basic cellular characteristics, including membrane potential, cell volume, and the state of intracellular signaling pathways. Thus, aberrant expression or function changes of ion channels may produce global or local, spatially restricted changes in these characteristics and driving the transformation of normal cells into malignant derivatives^8–11^. Some previous research also found that regulating of certain ion channels can inhibit the occurrence and development of tumor^12–16^. Beyond that, the expression level of transient receptor potential ion channel, subfamily V, member 4 (TRPV4) vary in different tumors and play important role in multiple processes of tumor progression. TRPV4 is a calcium-permeable nonselective cation channel of TRP family, which biologically involved in osmotic sensitivity and mechanosensitivity^17,18^. TRPV4 participates in many physiological processes, such as liver^19^, intestinal^20^, renal^21^ and bladder^22^ functions, growth and structural integrity of the skeleton^23^, together with systemic osmotic pressure induced by the brain^24^. Until now, TRPV4 has been reported functionally related to cell proliferation^25^, differentiation^26^, apoptosis^27^, migration^28^, and many other physiological processes. In addition, TRPV4 has also been significantly correlated with tumor angiogenesis^29^. Targeting TRPV4 may have inhibitory effects on tumor onset and progression, so it is a potential prognostic index and therapeutic target for malignant tumors. We herein reviewed the research focus on the relationship between TRPV4 and cancer, also explored the mechanism of TRPV4-mediated oncogenesis and the strategy that target TRPV4 for tumor metastasis. We hope this finding provide valuable reference for further research and clinical application.

## TRP superfamily

Similar to voltage-dependent cation channel, TRP channel also has six-transmembrane (S1–S6) domains, and both the N-terminus and C-terminus are intracellular. The segment between S5 and S6 of the TRP channel is embedded to form an ion-passage channel^30^. The S4 fragment lacks positively charged amino acid residues of that in normal voltage-dependent cation channel, i.e., the TRP channel is nonvoltage dependent. Besides, there are several ankyrin (ANK) repeat domains at the N-terminus of many TRP subtypes^31^. According to the amino acid sequence homology, more than 30 mammalian TRP channels have been classified into seven subfamilies: TRPC, TRPV, TRPM, TRPA, TRPN, TRPML, and TRPP^32^. The TRP channel is a nonselective cation channel on the cell membrane through which calcium and sodium ions mainly pass^33^. However, the ratios of selectivity of calcium ion to that of sodium ion (PCa/PNa, P stand for permeability) are different, ranging from higher than 100:1 to lower than 0.05:1^34,35^. In addition, many divalent ions, such as magnesium (Mg^2+^), zinc (Zn^2+^), manganese (Mn^2+^), and cobalt (Co^2+^), can also pass through TRPM6 and TRPM7 channels^36–38^.

As a crucial sensor of cells, TRP channels can transmit information between intracellular and extracellular, also being regulated by changes in messenger molecules, compounds, temperature, and osmotic pressure^39^. Different TRP channels have various regulatory mechanisms, many of which are modulated by a variety of stimulations. Although the activation mechanisms of different TRP channels vary, they can be roughly classified into receptor activation, ligand activation, and temperature-change activation. G protein-coupled receptors or tyrosine kinase receptors can activate TRP channels by activating phospholipase C (PLC)^40,41^. Some exogenous small molecules, compounds, inorganic ions, and endogenous substances can also activate these channels^42^. Temperature changes directly activate multiple TRP channels, and the activation thresholds of different temperature-sensitive TRP channels vary^43^. TRPV4 is activated by moderate heat (>24–27 °C), while TRPA1 is activated by noxious cold at 17 °C and below. TRP channel is widely distributed in the peripheral nervous system, skin, cardiovascular system, respiratory system, gastrointestinal system, genitourinary system, and immune systems in addition to the central nervous system^44–48^. Unlike the classical voltage-dependent cation channel that participates in specific functions, TRP channel has a variety of functions, mainly including temperature and pain perception^49^, gustation^50^, feeling of mechanical force^51^, mediation of PLC-dependent calcium influx^52^, maintenance of cell ion homeostasis^35^, as well as regulation of cell growth^53^, neurotransmitter release, and hormone secretion^54,55^.

Recently, several members of the TRP superfamily have been confirmed to play certain roles in tumor progression. Accumulating evidence has indicated that TRP members were expressed abundantly in cancer cells and closely related to tumor progression. Kim et al. reported that TRPM7 played a key role in the growth and survival of gastric cancer cells^56^. Elevated expression of TRPC5 protein is associated with the drug resistance of breast cancer cells^57^, and promotes colon cancer metastasis via the hypoxia-inducible factor 1-alpha/Twist (HIF-1α/Twist) signaling pathway^58^. Meanwhile, TRPV4 is aberrantly expressed in many tumors and strongly corelated with the prognosis of cancer. The different members of the TRP family and their main characteristics are summarized in Table 1, including the physiological functions, activation conditions, and their effects on cancer.

**Table 1:** Different members of the TRP family and their main characteristics

Channel subunit	Physiological functions	Activation	Effects on cancer	Refs.
*TRPC subfamily*				
TRPC1	Mechanosensation; salivary gland fluid secretion and appetite control; generation of the excitatory postsynaptic potential in brain; promotion of brain development (together with TRPC5)	Ca^2+^ store depletion stress; mechanical stretch; PLC signaling pathway	Promoted cell migration (H1080 cells)	127–129
TRPC2	Gender recognition and aggression behaviors signal transduction (mouse)	Diacylglycerol (DAG)	ND	127, 128,130
TRPC3	Regulation of cerebral vasomotor; guide of growth cones; spine formation in brain; motor behavior in the cerebellum	Ca^2+^ store depletion stress; DAG	Promoted melanoma cell viability and migration	131–133
TRPC4	Regulation of endothelium-dependent vasorelaxation; regulation of transcellular permeation of the endothelial layer and endothelium intercellular adhesion; participation in 5-HT signal transduction	GPCR and receptor tyrosine kinases and their downstream components; Ca^2+^ store depletion stress	Inhibited proliferation (SW982 cells)	134,135
TRPC5	Control of anxiety, fear, and reward behaviors; promotion of brain development (together with TRPC1)	GPCR and receptor tyrosine kinases and their downstream components; Ca^2+^ store depletion stress	Improved colorectal cancer, breast chemoresistance; associated with autophagy, inhibited proliferation	135–137
TRPC6	Redox sensor; regulation of artery contractility and angiogenesis; participation in endocannabinoid signal transduction; promotion of dendrite growth and synapse forming in the developing brain	DAG, PtdIns(4,5)P_2_ (PIP_2_)	Promoted breast proferation, migration	127, 128,138
TRPC7	Control respiratory rhythm activity in pre-Bötzinger complex in the brain; regulation of vasoconstriction	DAG, Ca^2+^ store depletion stress, GPCR	ND	128,139
*TRPV subfamily*				
TRPV1	Thermosensation (heat); autonomic thermos regulation; inflammatory hyperalgesia; regulation of osmosensing in the brain by a particular TRPV1 variant; nociception and pain management; endocannabinoid signaling in the brain; food intake signal regulation	Heat (>43 °C), vanilloids (capsaicin)	Promoted cell proliferation (PC3cells); promoted migration; induced cell apoptosis (U373 or HeLa cells)	140,141
TRPV2	Thermosensation (noxious heat); nociception; mediated immune response	Heat (>52 °C), osmotic cell swelling and LPLs	Induced apoptosis (T24 cells); promoted migration and invasion (﻿prostate cancer)	140,142
TRPV3	Thermosensation (moderate heat); nociception; skin integrity, hair growth and sebocyte function, mood regulation	Heat (>33 °C)	Promoted lung cancer proliferation	143,144
TRPV4	Thermosensation (moderate heat); mechanosensation; osmo-sensation; nociception; endothelium vasomotor control and shear stress sensor; modulation of cell migration; control adherens junctions in skin	Heat (>24-27°C); mechanical deformation and osmotic stimuli; PH; 5,6-EET	Destabilized cancer vascular integrity (﻿mouse prostate cancer) and promoted migration (breast cancer)	114,145
TRPV5	Ca^2+^ reabsorption in kidney epithelial cells; bone density	Activated via PLC; PH; osmotic stimuli	Promoted proliferation and metastasis (renal cell carcinoma)	140, 146,147
TRPV6	Ca^2+^ reabsorption channel in kidney; bone density; keratinocyte development in the skin; regulation of calcium entry into intestinal enterocyte	Constitutively active; osmotic stimuli; Ca^2+^ store depletion stress	Promoted proliferation (ovarian cancer)	148,149
*TRPM subfamily*				
TRPM1	Light-evoked response in ON bipolar retinal ganglia cells; regulation of melanin content in human epidermal melanocyte	Constitutively active	Reduced melanoma cells metastasis	127, 128,150
TRPM2	Thermosensation (moderate heat); oxidative and nitrosative stress response; immunity cells infiltration; regulation of pancreas insulin release; apoptosis control	Heat (>35 °C)	Promoted Migration, invasion (AGS cells, breast cancer) and promoted proliferation (prostate cancer)	15, 127, 128, 150,151
TRPM3	Regulation of pancreas insulin release and glucose homeostasis; steroid hormone (pregnanolon) sensor	Heat (>35 °C); cell swelling	ND	127, 128,152
TRPM4	Regulation of catecholamine release from chromafn cells; involved in mast cell activation and dendritic cell migration; regulation of Ca^2+^ entry	Heat (~40°C)	Induced EMT and promoted invasion, metastasis (prostate cancer)	127, 150,153
TRPM5	A key component of taste (sweet, bitter, umami) transduction; regulator of glucose-induced insulin release	Actived via GPCR	Inhabited metastasis (B16BL6 cells)	127, 154,155
TRPM6	A key component of taste (sweet, bitter, umami) transduction; positive regulator of glucose-induced insulin release; trigeminal nasal chemoreception	ND	Promoted proliferation (SHEP-21N cells)	127,156
TRPM7	Mg^2+^ homeostasis and reabsorption in kidney and intestine; development of thymocytes	Membrane stretch activated and Mg-ATP inhibited	Promoted migration and invasion (prostate cancer)	127,140
TRPM8	Thermosensation (cold); autonomic thermos regulation (with TRPV1)	Cooling (<28 °C), PIP_2_ and LPLs	Decreased migration (prostate cancer); increase apoptosis and oxidative stress (Du145 cells); promoted cancer viability (prostate cancer, pancreatic adenocarcinoma)	140,157–160
*TRPA1 subfamily*				
TRPA1	Thermosensation (noxious cold); mechanosensation; chemosensor; nociception; inflammatory pain	Heat (<=17 °C)	Anti-ROS induced cell death; induced autophagy and promoted invasion (lung cancer)	161,162
*TRPML subfamily*				
TRPML1	Essential for endocytosis and endosomal/lysosomal function; regulation of autophagy; regulation of neurological function	PIP_2_, pH	Promoted proliferation and invasion (triple-negative breast cancer)	163–165
TRPML2	Endosomal/lysosomal function	PIP_2_	Promoted proliferation and viability (glioma)	166,167
TRPML3	Endosomal/lysosomal function; autophagy; hair cell maturation; regulation of autophagy	PIP_2_	ND	168,169
*TRPP subfamily*				
TRPP2	Regulator of endogenous mechanosensitive channels; cardiac, skeletal and renal development; integrity of the vessel wall; mechanoreceptor and flow sensor in endothelium; apoptosis	EGF	Promoted EMT and invasion (squamous cell carcinoma)	170,171
TRPP3	Renal development; part of putative sour sensor	ND	ND	172,173
TRPP5	Spermatogenesis	ND	ND	174

*ND* not determined

## TRPV4

### Structure and function of TRPV4

**Fig. 1:**
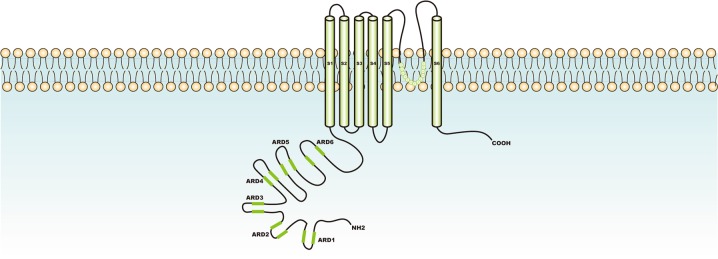
Structure of TRPV4. Similar to other transient potential receptor proteins, TRPV4 is consisted of 871 amino acids, has a homodimeric tetramer structure with six transmembrane spanning α-helices (S1–S6) per TRPV4 monomer

The TRPV family consists of six members (TRPV1–TRPV6), which all function as tetramers. Among them, TRPV1–TRPV4 have moderate permeabilities for calcium ions, with the PCa/PNa ratios of 1–10. With the ratios of over 100, TRPV5–TRPV6 are highly permeable for calcium ions^34^. The TRPV4 ion channel was described in detail dating back to 2000, and characterized as a volume-regulated channel due to osmotic sensitivity and cell volume regulation^59^. TRPV4 gene encodes TRPV4 ion channel protein, which was initially referred to as “vanilloid-receptor related osmotically activated channel” and “OSM9-like transient receptor potential channel, member 4”^60^, as a member of the vanilloid subfamily in the TRP superfamily^61^. TRPV4 channel protein consisting of 871 amino acids has a homodimeric tetramer structure which is similar to those of other transient potential receptor proteins, with six transmembrane spanning α-helices (S1–S6) per monomer^62^. The structure of TRPV4 is shown in Fig. 1. In addition to the transmembrane region, the remaining part of this protein is located in the cytoplasm. Similar to other TRPVs, it has six ANK repeats at the N-terminus, which are essential for the regular functioning of ion channels and protein–protein interactions. As a nonselective cation channel (Ca^2+^ or Mg^2+^ as the permeating extracellular cation), TRPV4 is characterized with a moderate high Ca^2+^ permeability ratio (PCa/PNa= 6–10, PMg/PNa = 2–3)^63,64^. The pore-forming loop that allows the ionic flow is located between S5 and S6 domains of TRPV4^65^. Some molecules, such as phosphatidylinositol 4,5-bisphosphate, can bind firmly to the ANK repeats end of TRPV4, thereby inhibiting the effects of TRPV4^66^. Temperature, mechanical force, hypotonia, phorbol ester derivatives, and other physical and chemical stimuli can activate TRPV4, allowing calcium-based cations to rapidly enter the cytoplasm to maintain osmotic pressure stability and signal transmission^66^. The representative agonists and antagonists of TRPV4 are organized in Table 2. TRPV4 is widely expressed in the nervous system^67^, immune system^68^, eye^69^, ear^70^, cardiovascular system^71^, respiratory system^72^, urinary system^73^, and digestive system^74^. Moreover, TRPV4 maintains osmotic pressure homeostasis by activating, rapidly and efficiently causing the influx of calcium-based cations, and maintaining cell morphology^75^. When skin tissue is physically and chemically stimulated, opening of the TRPV4 promotes the mechanical responses of subcutaneous fibroblasts and endothelial cells, manifested as vasodilation and skeletal muscle relaxation^76^. Different physical and chemical stimuli include heat, mechanical force, and endogenous substances, such as arachidonic acid and its cytochrome P450-derived metabolites (epoxyeicosatrienoic acids), endocannabinoids (anandamide and 2-arachidonoylglycerol), as well as synthetic a-phorbol derivatives can activate TRPV4. TRPV4 integrates multiple stimuli, then transmitting calcium signals and inducing a series of stress responses, such as promotion of release of nitric oxide, prostaglandin I2, and endothelial-derived enoic acid in the vascular endothelial system, relaxation of vascular smooth muscles, production of inflammatory factors (e.g., interleukin-6 (IL-6)) in lung tissue, and development of inflammatory responses^77,78^. At the early stage of vascular and neuronal development, activation of the TRPV4 channel of capillary endothelial cells and neurons activates downstream phosphatidylinositol 3-kinase (PI3K) and induces the activation of α-integrin protein, thereby facilitating the localization and remodeling of neurons and endothelial cells^79^. In adipocytes, TRPV4 is involved in fatty acid metabolism. Activating TRPV4 not only increases fatty acid synthesis by regulating RAC-alpha serine/threonine-protein kinase (AKT) phosphorylation but also attenuates fatty acid oxidation to reduce heat production^80^.

**Table 2:** Data summary for agonists and antagonists of TRPV4

Compound	Structure	Formula	Speciality	Species	Selectivity	EC50	Refs.
GSK1016790A		C_28_H_32_Cl_2_N_4_O_6_S_2_	Agonists	Human, mouse	Selecitve	hTRPV4: 2.1 nM mTRPV4: 18 nM	90, 175,176
4αPDD		C_40_H_64_O_8_	Agonists	Human, mouse, rat	Nonselective (channel unknown)	hTRPV4: 0.2 µM	175,177–179
Phorbol 12 myristate 13-acetate		C_36_H_56_O_8_	Agonists	Human, mouse	Nonselective (agonists of TRPV1)	hTRPV4: 11.7 nM	62, 175,176
N-arachidonoyl taurine		C_22_H_37_NO_4_S	Agonists	Mouse	Nonselective (agonists of TRPV1)	mTRPV4: 21 µM	180
5,6-EET		C_20_H_32_O_3_	Agonists	Mouse	Nonselective (inhibitor of T-channel Cav3 currents; agonists of TRPA1)	ND	181–185
Apigenin		C15H10O5	Agonists	Human, mouse, rat	ND	hTRPV4: 4.32 µM	186
Bisandrographolide A		C40H56O8	Agonists	Mouse	Selecitve	hTRPV4: 0.79-0.95 µM	181,187
Dimethylallyl pyrophosphate		C20H36O7P2	Agonists	Human, mouse	Nonselective (antagonists of TRPV3)	hTRPV4: 2.5 µM	188
Gd3+		Gd	Antagonists	Human, rat	Nonselective (antagonists of non-selective TRPVs channels)	ND	62, 189,190
La3+		La	Antagonists	Human, rat	Nonselective (antagonists of non-selective TRPVs channels)	ND	62, 189,190
Ruthenium red		H42Cl6N14O2Ru3	Antagonists	Human, rat	Nonselective (inhibitor of SERCA; antagonists of non-selective TRPVs channels)	ND	175, 189,191
HC-067047		C26H28F3N3O2	Antagonists	Mouse, rat	Selecitve	hTRPV4: 0.36 µM	106, 192,193
RN-1734		C14H22Cl2N2O2S	Antagonists	Human, mouse, rat	Selecitve	hTRPV4: 0.77 µM	192
Capsazepine		C19H21ClN2O2S	Antagonists	Human, rat	Nonselective (antagonists of TRPV1)	ND	192,194–196
GSK2193874		C37H38BrF3N4O	Antagonists	Human, mouse	Selective	hTRPV4: 0.65 µM	192, 197,198

*ND* not determined

### TRPV4 is involved in tumor onset and progression

**Fig. 2:**
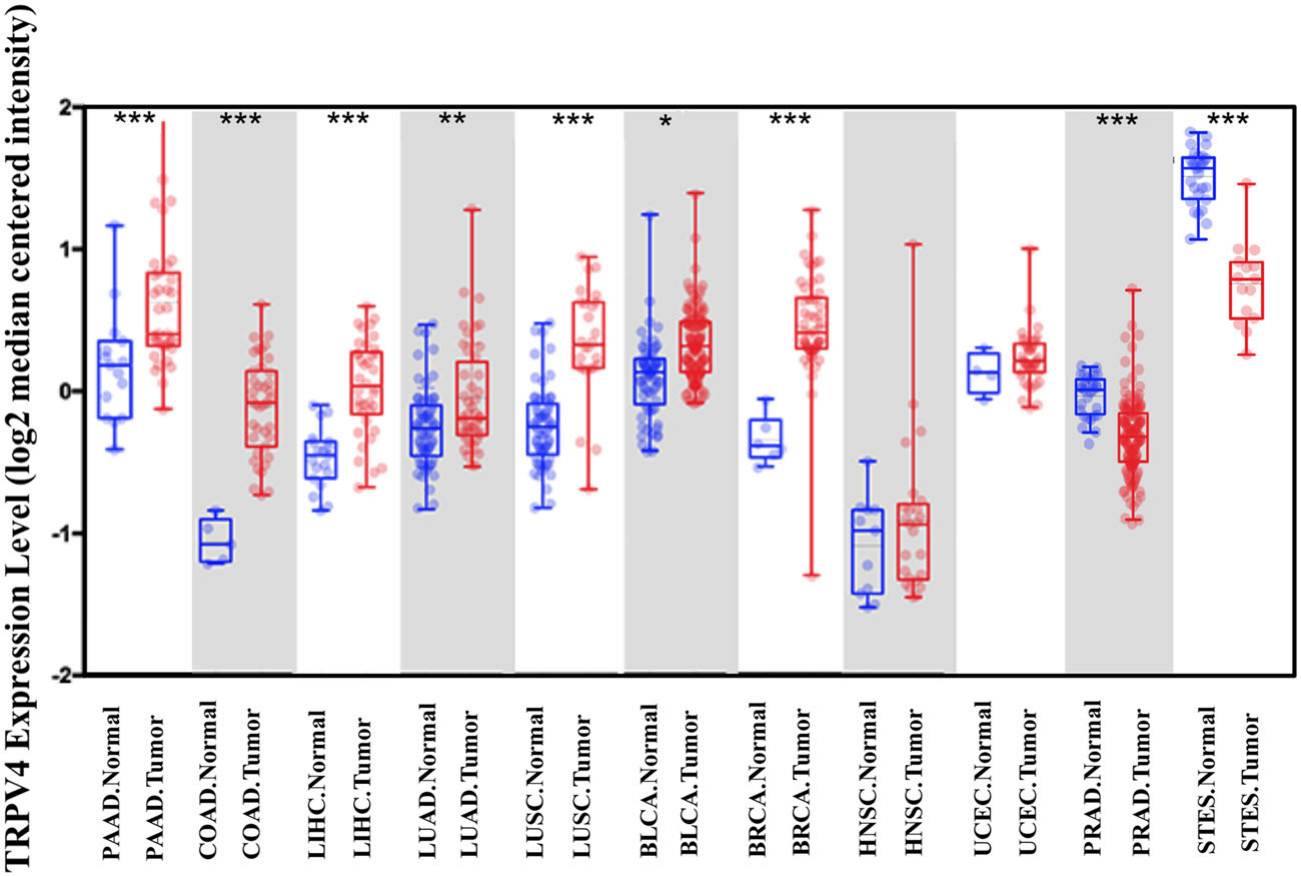
Expression of TRPV4 in human tumor samples obtained from oncomine. Data obtained from oncomine and the table are made by the data set from PAAD (pancreatic adenocarcinoma): series GSE16515 (title: FKBP51 affects cancer cell response to chemotherapy by negatively regulating Akt), with *p*-value of 8.93E-4, and 52 patients samples were used for this analysis; COAD (colon adenocarcinoma): series GSE5206 (title: Transcriptional recapitulation and subversion of embryonic colon development by mouse colon tumor models and human colon cancer), with *p*-value of 5.56E-7, and 46 patients samples were used for this analysis; LIHC (liver hepatocellular carcinoma): series GSE14323 (title: Genes involved in viral carcinogenesis and tumor initiation in Hepatitis C virus-induced hepatocellular carcinoma), with *p*-value of 1.36E-9, and 57 patients samples were used for this analysis; LUAD (lung adenocarcinoma): series GSE19188 (title: Gene expression-based classification of non-small cell lung carcinomas and survival prediction), with *p*-value of 0.002, and 110 patients samples were used for this analysis; LUSC (lung squamous cell carcinoma): series GSE19188 (title: Gene expression-based classification of non-small cell lung carcinomas and survival prediction), with *p*-value of 5.18E-9, and 92 patients samples were used for this analysis; BLCA (bladder urothelial carcinoma): series GSE13507 (title: Expression signature of E2F1 and its associated genes predict superficial to invasive progression of bladder tumors), with *p*-value of 0.035, and 130 patients samples were used for this analysis; BRCA (breast invasive carcinoma): series GSE9014 (Title: Stromal gene expression predicts clinical outcome in breast cancer), with *p*-value of 3.54E-7, and 59 patients samples were used for this analysis; HNSC (head-and-neck squamous cell carcinoma): series GSE7410 (title: Gene expression in early stage cervical cancer), with *p*-value of 0.908, and 54 patients samples were used for this analysis; UCEC (uterine corpus endometrial carcinoma): series GSE19188 (title: Fibroblast growth factor 9 has oncogenic activity and is a downstream target of Wnt signaling in ovarian endometrioid adenocarcinomas), with *p*-value of 0.067, and 45 patients samples were used for this analysis; PRAD (prostate adenocarcinoma): series GSE21034 (title: Whole-transcript and exon-level expression data for human primary and metastatic prostate cancer samples and control normal adjacent benign prostate), with *p*-value of 3.71E-12, and 160 patients samples were used for this analysis; STES (esophageal carcinoma): series GSE13898 (title: Robust prognostic biomarkers for EAC identified by systems-level characterization of tumor transcriptome), with *p*-value of 6.58E-9, and 40 patients samples were used for this analysis. We queried oncomine for each of those specific cancers to compare the fold change of TRPV4 in cancer tissue cells with normal tissue cells. All *p*-values represent a Student’s *t* test

Abnormal expression of TRPV4 is closely related to tumor formation and metastasis, which is higher in gastric cancer, lung cancer, and colorectal cancer cells, but lower in esophageal cancer and prostate cancer cells than in normal tissue cells according to the researches on TRPV4 (Table 3). The expression data of TRPV4 obtained from oncomine (https://www.oncomine.org/resource/main.html) show similar results (Fig. 2). Since TRPV4 can be activated at the condition of body temperature, its high expression in these cancer cells may led the intracellular calcium higher than other cells. Once TRPV4 is activated by some other stimuli, the rapidly increased intracellular calcium can regulate the downstream signaling pathway to affect the different processes of tumorigenesis. However, TRPV4 downregulation in cancers might be related with the differences in tumor microenvironment. Collectively, TRPV4 probably affect cell proliferation, differentiation, apoptosis, and migration by regulating Ca^2+^ and production of isoforms, thus affecting tumor onset and progression.

**Table 3:** Expression of TRPV4 in various cancer

Cancer type	Expression	Refs.
Gastric cancer	Up	88
Lung cancer	Up	199
Colorectal cancer	Up	200
Esophageal cance	Down	201–203
Prostate cancer	Down	204
Pancreatic cancer	Up	205
Liver cancer	Up	206

The results of lung cancer and colorectal cancer in this table and Fig. 2 are obtained from the same research

#### Role of TRPV4 in cell proliferation and differentiation

Proliferation and differentiation are two basic cell biological events. It is well-accepted that cancer cells require unlimited replicative potential to generate macroscopic tumors^81^. This capability is different from the behaviors of most normal cell lineages which have limited number of successive growth-and-division cycles^82^. Most cancer cells have been considered immortal after transformation from normal cells, as the starting point of tumorigenesis. TRPV4 may dominate in the process of proliferation, and it’s upregulation is strongly linked to the proliferation of hepatic stellate cells (HSCs). Song et al. reported that the TRPV4 mRNA and protein expression levels of rat HSC-T6 cell line after treatment with transforming growth factor β1 significantly exceeded those of control group. However, TGF-β1-induced HSC-T6 cell proliferation was inhibited by Ruthenium Red (a nonspecific inhibitor of TRPV4) or synthetic siRNA targeting TRPV4^83^. When TRPV4 activated, HSCs can be transformed into myofibroblasts which in turn secrete a large amount of collagen, leading to liver fibrosis, damage to liver tissue structure, cirrhosis, and eventually liver cancer^84,85^. Accordingly, increased expression of TRPV4 can indirectly induce liver cancer by stimulating the proliferation of HSCs. Functionally expressed in oligodendrocyte precursor cells (OPCs), TRPV4 can increase their proliferation. Ohashi et al. detected TRPV4 mRNA expressions in OPCs in vivo and primary cultured rat OPCs. Stimulating TRPV4 by GSK1016790A augmented OPC proliferation, which was abolished by co-treatment with HC-067047^86^. Given that OPCs are the origin of malignant glioma cells, overexpression or overactivation of TRPV4 may exert evident effects on the development of malignancies. Huang et al. showed that the overactivation of TRPV4 promoted the proliferation and/or migration of esophageal squamous cell carcinoma^87^. In addition, TRPV4 activation may selectively inhibits tumor endothelial cell proliferation via inhibition of ERK1/2 phosphorylation^25^. Studies have also shown that calcium-sensing receptor (CaSR) and TRPV4 were colocalized in gastric cancer cells, and CaSR activation evoked TRPV4-mediated Ca^2+^ entry promote gastric cancer cells proliferation^88^.

If a gene cannot be specifically expressed during maturation, the differentiation process is inhibited, which is primarily responsible for tumorigenesis. The growth and differentiation of keratinocytes are affected by intracellular and extracellular Ca^2+^ concentrations. When the extracellular Ca^2+^ concentration is low, primary keratinocytes remain undifferentiated. Conversely, cell proliferation is suppressed and differentiation is thus facilitated in the presence of high-concentration Ca^2+^^89^. TRPV4 is highly expressed in healthy or inflamed skin, whereas lowly or even not expressed in precancerous lesions and nonmelanoma skin cancer. In human keratinocytes, activation of TRPV4 stimulates the release of IL-8, which in turn downregulates TRPV4 expression^90,91^. Hypothetically, low expression of TRPV4 in skin cancer affects the release of ATP and autocrine communication between keratinocytes by regulating Ca^2+^ homeostasis, then decreasing extracellular Ca^2+^ concentration, keeping cells intact and ultimately inducing tumor formation^91^. In summary, TRPV4 plays an important role in the proliferation and differentiation of cells, which further affects the progression of cancer.

#### Role of TRPV4 in cell apoptosis

In multicellular organisms, the total number of cells is delicately balanced by the cell-generating effects of mitosis and cell death induced through apoptosis, which, when disrupted, results in cancer progression^92^. As a self-monitoring mechanism of organisms for hyperproliferative cells, apoptosis is also an effective means of tumor treatment. TRPV4 can inhibit the expressions of apoptotic proteins via a variety of (direct and indirect) pathways, and plays an indispensable role in suppressing apoptosis. Knockdown of TRPV4 expression by siRNA or pharmacological inhibition of TRPV4 can attenuate neuronal apoptosis^18^. Nonetheless, overactivation of TRPV4 can induce apoptosis. Activation of TRPV4 by GSK1016790A induces apoptosis by downregulating PI3K/Akt and upregulating p38 MAPK signaling pathways^93^. TRPV4 is a nonselective cation channel that works mainly based on channel switches. Zhan et al. treated HSC-T6 cells with 4α-phorbol 12,13-didecanoate (a TRPV4 activator) which then suppressed apoptosis and enhanced autophagy^94^. There are existing insights suggested substantial cytotoxicity of TRPV4 overactivation. GSK1016790A can cause strong calcium-overload and cellular disarrangement, increased the rate of apoptosis, and strongly inhibited human melanoma cell lines (A375, SK-MEL-28, MKTBR) proliferation/survival, similarly in HaCaT keratinocytes^90^, also in breast cancer cell line MDA-MB-468 pharmacological activation of TRPV4 produced pronounced cell death through apoptosis and oncosis^27^. It can be seen that activation of TRPV4 can induce the occurrence of apoptosis from above results. Classical calcium signaling pathway has manifested that calcium influx can regulate apoptosis by regulating caspase signaling pathway which was regulated by calpain. This may also be the main mechanism of TRPV4 in apoptosis.

#### Role of TRPV4 in tumor metastasis

It is well-documented that the expression level of TRPV4 in tumor cells was positively correlated with their metastatic ability. Lee et al. performed high-throughput sequencing for isogenic breast cancer cell lines, and found that the expression levels of TRPV4 in 4T07 and 4T1 cells which were prone to exudation and spread during metastasis were abnormally elevated^95^. Fusi et al. detected the expression of TRPV4 by immunohistochemical assay and found that the TRPV4 in weakly metastatic squamous cell carcinoma and basal cell epithelial cancer were significantly lower than that of normal skin tissue, whereas the expression in strongly metastatic malignant melanoma was higher than that of normal tissue^89^. Mrkonjić et al. transfected HEK293 cells with TRPV4. As a result, the cell migration ability was significantly enhanced compared with that of the blank control group. After transfecting with TRPV4 lacking a phosphoinositide-binding site, TRPV4 of HEK293 cell opens continuously which allowing further augmentation of cell motility and directionality^96^. Tumor-related death has mainly been attributed to metastasis^97^. Taken together, TRPV4 is abnormally highly expressed in many tumors which significantly correlated with their metastasis potential. Hence, it may be a therapeutic target for inhibiting tumor metastasis.

## Influence of TRPV4 on tumor metastasis and possible mechanism

Regardless of significant advances achieved in research, diagnosis, and treatment of cancers, the vast majority of advanced metastatic cases, with rare exception, cannot be cured by current regimens. Being closely related to the prognosis of tumor patients, TRPV4 is probably one of the main participants in tumor metastasis. Therefore, it is of great significance to clarify the mechanism of TRPV4 in tumor metastasis.

### TRPV4 promotes epithelial–mesenchymal transition (EMT)

EMT confers on tumor cells properties that are critical to invasion and metastatic dissemination, notably increased motility, invasiveness, and degradation of components of the extracellular matrix. The cells are transformed into spindle-shaped fibroblast-like ones, accompanied by enhanced motility^98^. TRPV4 can mediate the occurrence of EMT. E-cadherin is a key inhibitor of neonatal epithelial tumor cell movement and invasion, thus the decreased expression of E-cadherin is considered to be a significant marker of EMT^99^. TRPV4 is highly expressed in bladder cancer cells, which, when suppressed, can significantly downregulate the expression of E-cadherin. Meanwhile, inhibiting TRPV4 can induce the activation of AKT and FAK to further alter the expression level of E-cadherin^100^. Cell motility enhancement is typified by cytoskeletal protein remodeling, also as a crucial feature of EMT^101,102^. In mammal cells, TRPV4 and cytoskeletal protein actin have extensive colocalized expression. Breast cancer cells in which TRPV4 is activated undergo cytoskeletal remodeling. As an upstream “signal emitter”, TRPV4 regulates not only microtubule–microfilament polymerization but also the dynamic changes of microvilli, filopodia, and slab pseudopods, thereby affecting cell motility^103^. The release of many factors in the tumor microenvironment, such as epidermal growth factor (EGF), tumor necrosis factor-α (TNF-α), and signal transducer and activator of transcription 3 (STAT3), can induce the occurrence of tumor EMT, and these factors are regulated by intracellular calcium signaling^104^. Calcium ion chelators can inhibit the activation of EGF transduction signals and STAT3 in breast cancer cells^105^. Furthermore, the levels of calcium ions in tumor epithelioid cells and mesenchymal cells are different^62^. Raising calcium concentration can induce EMT of breast cancer MDA-MB-468 cells, probably by mediating their morphological changes^105^. Using the specific antagonist HC-067047 to inhibit TRPV4 in hepatocellular carcinoma cells suppressed cell proliferation, induced apoptosis, and decreased the migration capability by attenuating the EMT process in vitro and intraperitoneal injection of HC-067047 could obviously suppress tumor growth in NOD-SCID mouse xenograft models^106^. Since TRPV4 is an essential ion channelregulating cell morphology, it may predominantly regulate EMT. TRPV4-mediated calcium signaling participates in the EMT process via multiple signaling pathways, including the Wnt/β-catenin and PI3K/AKT pathway. As to gastric cancer metastasis, TRPV4 can enhance PI3K/AKT activity upon activation, induce β-catenin to enter the nucleus, and activate EMT by interacting with nuclear transcription factor T lymphocyte factor/lymphoid enhancer^88^.

### TRPV4 promotes expressions of tumor metastasis-associated proteins

The promotive effects of TRPV4 on the EMT process have been elucidated above. When TRPV4 is continuously activated, the expressions of genes that facilitate tumor metastasis increase, but those of genes that inhibit metastasis reduce. Lee et al. found that the expressions of cell adhesion-associated tumor suppressor genes in mouse breast cancer cell line 4T07, such as Fn1, Clu, Tubb2c, and Spp1, decreased after administration with TRPV4 agonist 4α-PDD. At the same time, the expression of tumor metastasis-promoting gene Talin secreted by exosomes increased. The Kaplan–Meier survival analysis of 4142 patients with breast cancer revealed a significant positive correlation between Talin gene expression and lymphatic metastasis^95^. In the meantime, tumor cells released metastasis-promoting factors through exosomes to mediate tumor metastasis^28^. As the main histological barrier of tumor metastasis, ECM comprises collagen, glycoprotein, proteoglycan, and other components^107^. TRPV4 seems to be colocalized with F-actin in highly dynamic membrane structures, such as filopodia, microvilli, and lamellipodia edges. The interaction between TRPV4 and F-actin may be necessary for the activation of TRPV4 by hypotonic cell swelling, and disrupting the F-actin structure abolishes TRPV4-actin co-localization and leads to the loss of hypotonicity-induced Ca^2+^ influx and RVD^108^. Moreover, matrix metalloproteinases (MMPs) are important enzymes for the degradation of ECM^109^. Villalta et al. evaluated the effects of TRPV4 on mouse brain edema, and found that the expression of MMP2/MMP9 in the hippocampus was significantly inhibited by using HC-067046, a TRPV4 antagonist^110^. Furthermore, MMP2/MMP9 is associated with the metastasis of many types of cancers such as lung cancer^111^. Microtubule-associated protein 7 (MAP7) interacts with residues 798–809 at the C-terminus of TRPV4, possibly by increasing the expression of TRPV4 in the plasma membrane and linking the channel to cytoskeletal microtubules, forming a mechanosensitive molecular complex^112^. VPAC1, a member of the G protein-coupled receptor (GPCR) superfamily, is mainly activated by vasoactive intestinal peptide (VIP). Previous studies reveal that VPAC1 activation promotes migration and invasion of GC cells through TRPV4 channel-dependent Ca^2+^ entry, which in turn augments VIP expression. VIP significantly increased the lung metastasis of gastric cancer cells in vivo through the VPAC1/TRPV4/Ca^2+^ signaling axis^113^. In vivo angiogenesis assay demonstrated that TRPV4 regulates tumor vessel integrity by maintaining VE-cadherin expression at cell–cell contacts^114^.

### Regulatory effects of TRPV4 on tumor angiogenesis

Access to the host vascular system and generation of tumor blood supply are important processes in tumor progression. A large number of new blood vessels not only provide sufficient nutrients for promoting the continuous growth of tumors but also drive tumor spread and metastasis^115^. TRPV4 is critical for tumor angiogenesis, but it may affect various tumors differently. Under normal conditions, activation of TRPV4-mediated Ca^2+^ entry is translated into a pro-angiogenic signal by several decoders, such as the Ca^2+^-dependent nuclear factor of activated T cells, cytoplasmic 1 (NFATc1), myocyte enhancer factor 2C (MEF2C), and Kv channel interacting protein 3, calsenilin (KCNIP3/CSEN/DREAM), which drive endothelial cell proliferation, β-integrin, and PI3K, which promote endothelial cell motility^116^. When TRPV4 expression is subjected to interference with shRNA or an inhibitor is used, retinal vascular cells fail to form tubules, indicating that TRPV4 significantly participates in retinal vascular endothelial cell migration and tubule formation^117^. The functions of TRPV4 protein from breast cancer-derived endothelial cells are similar to those of normal endothelial cells. Activating TRPV4 by using arachidonic acid can significantly promote the migration of endothelial cells derived from human breast cancer, but not that of normal human microvascular endothelial cells, which may be ascribed to the significantly higher expression of TRPV4 in breast cancer. As a result, intracellular calcium increased to promote endothelial cell migration. In contrast, loss of TRPV4 expression inhibits arachidonic acid-induced breast cancer cell migration^118^. For lung cancer and prostate cancer, the expression levels of TRPV4 in tumor-derived endothelial cells are lower than those of normal endothelial cells, thus elevating the sensitivity of endothelial cells to extracellular matrix stiffness and being conducive to the formation of abnormal blood vessels^29^. In TRPV4 knockout mice, the density and diameter of new tumor blood vessels enlarge, and the coverage of surrounding tumor capillary endothelial cells shrinks. Therefore, the formation of blood vessels is “abnormalized” to further promote the progression of lung cancer. On the contrary, activating TRPV4 with GSK1016790A can “normalize” the vascular endothelium, enhance the permeability of chemotherapeutic drugs, and ultimately reduce the exudation of cancer cells and block tumor growth^119^. With the study mentioned before, these results demonstrate the tumor angiogenesis regulatory role of TRPV4. In short, inhibition of tumor angiogenesis by targeting TRPV4 should be based on a thorough understanding of the organ specificity of the tumor microenvironment. Nevertheless, the underlying mechanisms require further in-depth studies.

### TRPV4 interacts with tumor microenvironment to promote tumor metastasis

The tumor microenvironment is characterized by hypoxia, low pH, and high tissue pressure, often accompanied by inflammatory reactions, which preferentially selects more invasive and aggressive tumor cells and also impedes the tumor-killing action of immune cells^120^. Proinflammatory cytokines in the inflammatory microenvironment recruit and hijack immune cells to aid the immune escape of tumor cells^121^. These inflammatory factors also stimulate blood vessels and lymphatic vessels to change their permeability to increase protein and cell leakage, ultimately contributing to tumor metastasis^122^. TRPV4 is crucial throughout inflammation. At the initial stage of inflammation, cathepsin produced by the microenvironment of tumor inflammation can simultaneously activate TRPV4 and proteinase 2 which then synergize to continue the development of inflammatory response^123^. Meanwhile, TRPV4 is an essential effector protein that mediates inflammation and signal transduction. Inflammatory factors released in the tumor microenvironment, such as IL-1, IL-8, arachidonic acid, and phosphatase A2, as well as interstitial plasticity changes can promote opening of the tumor TRPV4, at which point the inflammatory factor release is reinforced by cascade amplification. Walter et al. reported that the expressions of inflammatory factors IL-1β, IL-6, and IL-8 decreased when intervertebral disc cells were inhibited by TRPV4^124^. Besides, Kim et al. found that inflammatory factors, such as TNF-α and IL-6, promoted the metastasis of Lewis lung cancer by increasing toll-like receptor 2 (TLR2) and its community toll-like receptor 6 (TLR6)^125^. When inflammatory response occurs, inflammatory factors recruit immune cells, and activation of the TRPV4 channel therein enables tumor cells to escape from the immune system. Scheraga et al. verified that activating the TRPV4 in bone marrow-derived macrophages was beneficial to the release of inflammatory factors, such as IL-1β and IL-10. Furthermore, activation of the TRPV4 of T cells at the site of inflammation facilitates the release of interferon-ɣ which is also an important mediator of tumor immune escape^126^. Collectively, targeting TRPV4 can simultaneously act on tumor cells and their “hijacked” immune cells, reduce the production of inflammatory factors, and finally ameliorate the inflammatory microenvironment for tumor metastasis.

## Conclusion

**Fig. 3:**
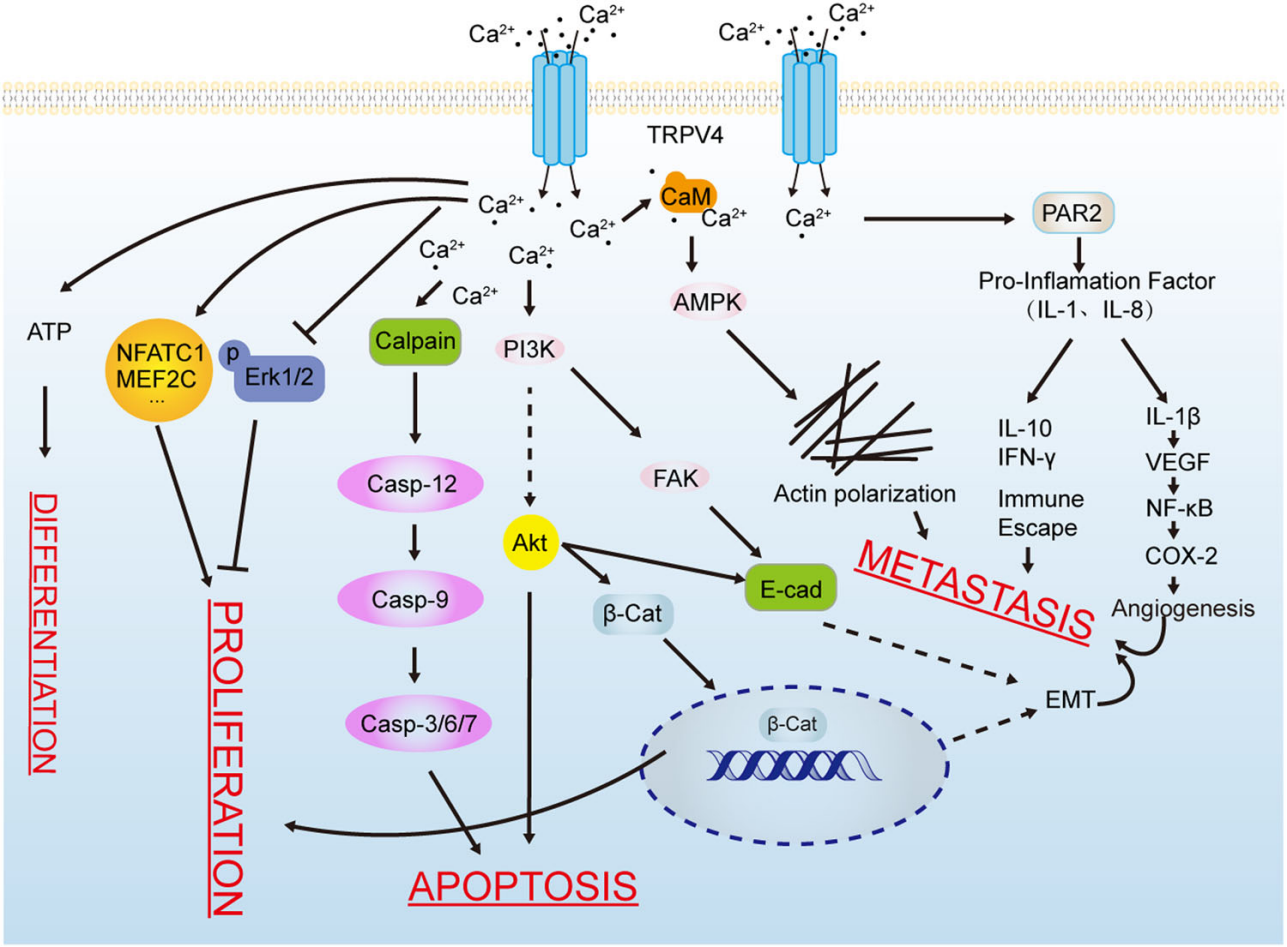
The specific process of TRPV4 acting on tumor occurrence and development and its possible mechanism. TRPV4 is closely related with the proliferation, differentiation, apoptosis, and migration of tumor cell by regulation of Ca^2+^ and production of isoforms, and finally participates in tumor onset and progression

In summary, this review clarified the roles of TRPV4 in tumor onset, progression, and metastasis together with the mechanisms, verifying its potential antitumor effects. The specific process of TRPV4 acting on tumor occurrence and development and its possible mechanism is shown in Fig. 3. It is explicitly that the expression and function of TRPV4 are closely related to the occurrence and development of tumors. Hence, TRPV4 is worthy of extensive research for the diagnosis, treatment, and prognosis of tumors. In recent years, the remarkable effects of calcium signal and relative channel protein on the progression of tumors have gradually been recognized. Cancer therapy can be improved by evaluating channel protein expression feature of different tumors then rationally selecting specific antagonists or agonists. Although TRPV4 inhibitors have been employed to treat various diseases such as pulmonary edema and heart failure, there are no commercially available low-toxic and efficient drugs hitherto. Furthermore, the functions of TRPV4 in tumors varied depending on the original tissue type. TRPV4 regulates cellular function by modulating calcium signaling, and finally participates in tumor onset and progression. As summarized above, TRPV4 is closely related with proliferation, differentiation, apoptosis, and migration of tumor cell by regulation of Ca^2+^ and its downstream, then finally participates in tumor onset and progression. Consequently, TRPV4 could be a potential therapeutic target of cancer treatment, thereby providing a new direction that develop drugs for cancer to reach the clinic. Regardless of burgeoning studies on the roles of TRPV4 in tumors, its differential expressions in different tumor tissues and the underlying mechanisms are still largely unknown. Therefore, developing antitumor drug targeting TRPV4 remains rather challenging. In addition, targeting TRPV4 channels may also affect other stromal cells, so it is imperative to assess the overall biological effects before possible clinical use.
